# Iranian EFL Teachers’ Emotional Labour and Grit as Predictors of Teacher Success

**DOI:** 10.11621/pir.2025.0304

**Published:** 2025-09-30

**Authors:** Mansoor Ganji, Farzad Sabeki

**Affiliations:** a Chabahar Maritime University, Iran

**Keywords:** emotional labour, grit, positive psychology, teacher effectiveness, teacher personality, teacher success

## Abstract

**Background:**

There is an absence of research – in EFL contexts – into the importance of positive psychology, notably the significant role of emotions in teaching. The topic of an instructor’s emotional labour and grit is particularly important in this context.

**Objective:**

The central focus of the current investigation was to determine the degree of association (predictive link) between teachers’ emotional labour and teacher grit, which represents the perseverance and passion for enduring objectives in the context of second language teaching with teacher success. Specifically, it sought to uncover the unique power of EFL teachers’ emotional labour and their grit in predicting the degree of their success in the Iranian context.

**Design:**

For this purpose, a quantitative approach with correlational design was adopted, and three questionnaires namely, Teacher Emotional Labour Strategies Scale (TELSS), Teacher Grit Scale, and EFL Language Teachers’ Effectiveness Questionnaire were administered to a sample of 184 English teachers across three different teaching contexts comprising Iranian private language institutes, public high schools, and universities in various provinces of Iran.

**Results:**

Results of regression analysis indicated that teacher grit is a significant and better predictor of teacher success compared to teachers’ emotional labour. Furthermore, the results of correlation analysis also confirmed that there was a positive relationship between teacher emotional labour, teacher grit and teacher success.

**Conclusion:**

The findings suggest that the grittier English teachers are, the more effective they are in their professional duties and work environments. Finally, theoretical and pedagogical implications are discussed, and several suggestions for future research are given.

## Introduction

Generally, a multitude of elements support the prosperity and success of every activity or enterprise. Among them, manpower is considered a very crucial factor in the success of any organisation. In educational contexts, teachers are considered the single most important element, and their characteristics and qualities can affect the success or failure of any enterprise (Coombe, 2019; Derakhshan & Shakki, 2019; Fan & Wang, 2022). This might be the reason numerous scholars have scrutinised the notions of teacher effectiveness as well as teacher success from various angles to provide a comprehensive picture of these concepts (Derakhshan et al., 2020). According to Wossenie (2014), the professional success of any teacher depends on how effectively they achieve the instructional goals established by either themselves or educational administrators. In the realm of foreign language education, the professional success of instructors is defined as the degree to which an English as a Foreign Language (EFL) instructor effectively imparts knowledge and skills related to the English to their language learners (Coombe, 2014).

Since the employment of productive and successful teachers is vital to the prosperity of any academic system and central to achieving the demanded learning outcomes (Coombe, 2019), factors affecting teachers’ career-related achievements and success are worth researching. To give an appropriate response to this need, dozens of researchers have examined the impact of personal and emotional factors in teacher’s professional success. These factors include: identity (Nazari et al., 2023a; Richards, 2023), autonomy (Chong, 2023; Nazari et al., 2023b), ambiguity tolerance (Ertürk et al., 2023; Huang, 2022; Sokolová & Andreánska, 2019), self-efficacy (Farkhani et al., 2022; Zhang et al., 2023), emotional intelligence (Kang, 2022; Zhi et al., 2023), professional development (Averina & Kuswandono, 2023; Gu et al., 2022), and credibility (Pishghadam et al., 2023; Sarani et al., 2020). However, this study seeks to provide a greater understanding into the influence of teachers’ emotional labour and grit on the professional success of teachers, which has been given scant attention by the former scholars.

As one of the factors affecting teachers’ success, the phrase “emotional labour” was first coined by the well-known sociologist Arlie Hochschild in 1983 and has gained more importance, particularly in the present century. Emotional labour is simply defined as exhibiting particular emotions to fulfil the expectations associated with a particular occupation. Hochschild (1983, p.7) refers to emotional labour as “the regulation of emotions to produce a visible expression through facial and physical gestures”. Benesch (2017, p.53), however, linked the notion of teacher emotional labour to English language teaching, describing it as “the process through which English language educators embark on self-regulation of their emotional expressions in accordance with bureaucratic policies and professional standards”. In light of the foregoing, emotional labour is perceived to be highly associated with interaction and teaching, managed and directed by cultural and social beliefs. It is worth mentioning that scholars interested in emotional labour have focused on other occupations and contexts, such as commerce and hospitality (Yang & Chen, 2021). Investigation of emotional labour in education seems to be highly important since the necessity for regular communication is a defining characteristic of the profession (Wróbel, 2013) but it has been under-researched. The management of this labour, the suppression, faking, or evoking of specifić emotions can be psychologically taxing and necessitates personal resources that allow educators to persevere in this demanding aspect of their work. In other words, teachers’ grit is one of the main sources which can help them manage their emotions because their perseverance helps them endure the emotional strain without burning out.

It is in this context that the concept of grit has garnered significant interest over the last decades from scholars in personality as well as cognitive and educational psychology (Derakhshan et al., 2025; Derakhshan & Fathi, 2024a; Derakhshan & Fathi, 2024b). As an approximately new concept, it is a prerequisite for educational achievement, success, and progress. Various fields such as business (Al Issa, 2020; Aronovitch & Gibaldi, 2023; de Waal et al., 2023) and healthcare (Lee et al., 2023; Samarasekera & Gwee, 2020; Traino et al., 2019) have also addressed teachers’ grit and have found it to be an important variable. Duckworth et al. (2007) emphasised this noncognitive element and explained it as “perseverance and passion for long-term goals” (p. 1087). Teacher grit also addresses similar qualities such as persistence and passion toward the attainment of prolonged objectives, however through the lens of educators (McCain, 2017). The prominence of teacher grit in instructional settings has been emphasied by Kazemkhah Hasankiadeh and Azari Noughabi (2023).

Besides research on teacher grit, a considerable body of research has focused in the last few years on various cognitive, behavioural, personality, affective, and classroom management characteristics of successful teachers (Al-Obaydi et al., 2023; Lee & Xie, 2023; Marashi, 2023). Yet little empirical investigation has been undertaken focusing on the contributions of instructors’ emotional labour and grit. Furthermore, very few studies have been conducted examining the role of these concepts simultaneously. As an attempt to fill this gap, this study has been designed to probe the contributions of teachers’ emotional labour and grit in order to illuminate on these concepts and their potential impacts on teacher success.

## Review of Literature

### Teacher Emotional Labour

One of the main variables of the study is emotional labour, which is characterised as the attempts, preparation, and leadership necessary for instructors to disclose authoritative necessary emotions in their bilateral interactions with others in the learning space and instructional environment (Yin et al., 2017). Hence, it is inevitable that communication with students will produce emotional labour for teachers. Besides, teachers’ emotions and behaviours are being constrained by organisational and societal norms and expectations. As a multidimensional construct, emotional labour portrays different processes such as emotion management and regulation, emotion dissonance, and displayed emotions at the workplace (Sarraf et al., 2017). Primarily, job-focused and employee-focused classification of emotional labour was suggested by Brotheridge and Grandey (2002). The first one targets occupational characteristics appertaining to emotional labour, namely the number of customer interactions, variety, display rules, duration, and intensity of emotions necessary within the framework of occupational communications. The second one pinpoints the emotional regulation processes such as management, effort and control, and internal states of the workers in performing emotional labour.

Earlier research on emotional labour (Li et al., 2012; Yin, 2012) showcased that instructors display emotional labour using deep acting, surface acting, and expression of naturally experienced emotion. Another well-established division of emotional lab our laid out by several scholars (Li et al., 2012; Noor & Zainuddin, 2011; Yin, 2012) suggests that the emotional labour performed by teachers is evident through three key mechanisms such as deep acting, surface acting, and the articulation of emotions that are genuinely felt. Surface acting is in connection with an approach whereby teachers “evoke emotions that are not experienced or alter the external manifestation of emotions that are genuinely felt” (Cukur, 2009, p. 561). Hence, deep acting refers to “the process of emotion regulation in which instructors strive to alter their experienced emotions using some cognitive procedures” to show required sensation and manner (Yin, 2012, p. 452). It is noteworthy that, surface as well as deep acting provides evidence for the instructors’ endeavours to be in line with emotional regulations and the void between experienced and exhibited feelings. Finally, the last strategy refers to the communication of authentic feelings that are consistent with prevailing emotional standards (Li & Liu, 2021).

Burić and Frenzel (2021) investigated the association among instructors’ emotional labour, recognised pedagogical approaches, and pupils’ self-reported engagement levels in the Croatian context. The research demonstrated that if educators did not unveil their original feelings in their instructional atmosphere, students noticed and benefited from their teaching strategies to a lower degree. Furthermore, engagement in the class was highly associated with teachers’ fake emotions. In a more recent qualitative study, Kang (2022) researched EFL teachers’ emotional labour and emotional intelligence. After classroom observation in a nonparticipant way, she reported that the instructor’s emotional intelligence along with emotional labour techniques were not constant in a semester. In the initial part of the semester, for instance, the teacher concentrated on the emotional labour in the form of inhibiting or maintaining emotions in addition to intrapersonal component of her emotional intelligence. In a recent attempt in the Iranian context, Nazari and Karimpour (2022) studied how emotional labour influences the identity formation of Iranian EFL teachers, utilising an activity theory framework. It was observed that the top-down approach in education resulted in tensions for educators, as they grappled with their internal perceptions and the diverse identities they embraced. Tahami Zarandi et al. (2023) in a cross-sectional survey, examined Iranian and Iraqi EFL teachers’ emotional labour, regulation as well as psychological well-being. They reported that in both countries, emotion regulation techniques and emotional labour strategies enjoyed high positive correlations with teachers’ well-being. Interestingly, Iranian EFL teachers, compared to their Iraqi counterparts, used more emotional labour strategies.

### Teacher Grit

The second variable which is hypothesised to affect teacher success is the notion of teacher grit. It has been regarded as a vital element and attracted much recognition in the realm of educational literature because of its positive relationship with pedagogical achievements (Santana-Monagas & Núñez, 2022; Shao, 2023). It has been characterised as coalescence of perseverance and passion regarding obtaining long-lasting objectives (Teimouri et al., 2022), and it provides assistance to the individuals surmount barriers and obstacles without resignation or experiencing burnout (Duckworth et al., 2007). As a complex parameter, grit has two major subcomponents: perseverance of effort and consistency of interest (Wang et al., 2021). Working hard and putting in more effort when hardships arise is referred to as perseverance of effort, and keeping one’s curiosity in obtaining objectives despite the presence of some setbacks is regarded as consistency of interest. Grit has been repeatedly proved to have a two-factor construction, and a small number of studies have found it as a single-dimensional construct (Postigo et al., 2021). This points towards further queries concerning the transparency of grit as a major and pivotal variable. Also, Duckworth et al. (2021) have also addressed this controversial issue and highlighted the role of environment in which it is being measured while interpreting the grit scale.

Several investigations have addressed the significance of teacher grit in instructional settings. Lee (2022) found that out of grit sub-scales, only perseverance of efforts along with classroom enjoyment are predictors of willingness to communicate between Korean EFL learners. In the Chinese context, it was found that learners’ grit could be increased through teacher respect and teacher support, thus EFL teachers are suggested to make their classrooms a supportive atmosphere so that their language learners become grittier (Shen & Guo, 2022). According to Shao (2023), teacher grit serves as a crucial mediator in the relationship between teacher well-being and motivation and engagement, which can contribute to its enhancement. In the Iranian EFL context, Fathi et al. (2021) reported that foreign language anxiety had a greater impact on willingness to communicate than grit. In another well-established attempt, Derakhshan et al. (2022) explored the relationship between several variables, including resilience, grit, and well-being, and their impact on the enjoyment experienced by Iranian EFL teachers in foreign language instruction. They observed that, besides teacher grit which was the most important predictor of foreign language teaching enjoyment, happy and resilient teachers were in most cases probable to have a strong positive mindset toward instructional experience in educational settings. Kazemkhah Hasankiadeh and Azari Noughabi (2023) showed that Iranian English language educators’ self-efficacious approaches could impact their grit to keep their resolution and enthusiasm for the profession.

### Teacher Success

The last concept which needs to be discussed here is teacher success, that is the level to which a teacher demonstrates effectiveness in the work setting (Elizabeth et al., 2008). Teachers’ success and its comprising qualities have gained significant attention in educational research recently (Coombe, 2014, 2019; Derakhshan et al., 2020; Pishghadam et al., 2019). Derakhshan et al. (2020) maintain that successful instructors are skilful, knowledgeable, and principled individuals who provide information in a very comprehensible way, apply unique instructional methods, and use several instructional aids. Bremner (2019) believes the characteristics of successful teachers are not restricted to their teaching performance. In his view, successful instructors are those who are able to create and keep language learners in a supportive and joyful learning atmosphere, care about their academic interests and well-being, and maintain an intimate relationship with them. In a similar study, Tamblyn (2000) identified qualities of successful instructors as possessing a sense of humour, showing respect towards students, possessing enough content knowledge, and finally being proud of their profession as an instructor. Furthermore, Coombe (2014, p.2) identifies ten characteristics that define highly effective and successful educators. She does not prioritise these qualities; however, she highlights the first one, which is having a “calling to the profession”. It refers to “the teachers who are driven and enthusiastic about what they do and feel a “call” to teach as well a passion to help their language learners learn and grow”. Fan and Wang (2022) in China, showed that well-being and emotion regulation had a strong connection with occupational success among EFL teachers.

Recently, Afsharpour et al. (2024) investigated the role of strictness and gender identity among EFL teachers. The results showed that the strictness of teachers has no bearing on the success rates associated with their gender, while gender identity and stroke had a great impact on predicting both male and female instructors’ success. Likewise, Derakhshan et al. (2020) utilised the structural equation modelling technique (SEM) to assess the impact of instructors’ professional identity and their degree of autonomy on achieving success within the educational landscape. The research indicated that both elements of teachers’ professional identity and their autonomy are significant factors in achieving overall success. In another attempt, Tabatabaee-Yazdi et al. (2018) examined Iranian EFL educators’ success rates in private language institutes according to language learners’ viewpoints. Their results pinpointed that an effective teacher should be able to make good decisions. SoodmandAfshar and Doosti (2014) using a validated open-ended questionnaire found that both students and teachers had similar perspectives regarding successful EFL teachers’ qualities. Both groups reported an effective instructor should be capable of conveying knowledge appropriately, knowing the subject matter, including all students in pair or group work tasks, having regular assessment plans, and being equipped with proper interpersonal relationships. Following the examination of the literature and the recognition of gaps in the topic, the research question presented below has been put forward:

Do Iranian EFL teachers’ grit and emotional labour significantly predict their success?

## Methods

### Participants

A quantitative approach was adopted for the current study. Consequently, snowball sampling was utilised to gather the quantitative data in the first phase. In snowball or network sampling, targetted participants were selected to recommend the names of others suitable for the sample (Ary et al., 2018). The researchers asked their colleagues and English teachers, with whom they had connections in various cities in Iran, to complete the questionnaires. These teachers were then asked to distribute the questionnaire link with other teachers they know.

Furthermore, convenience and volunteer sampling method were also used to enhance the sample size. The Google Forms link was distributed among English teachers in several Telegram groups to facilitate the data collection procedure. Overall, 184 English teachers participated in the study, including males (*n* = 51) and females (*n* = 133). They possessed BA (*n* = 50), MA (*n* = 97) and PhD (*n* = 37) degrees and had 1 to 27 years of teaching experience. Participants’ ages varied between 20 and 59 (*M* = 31, *SD* = 7.35).

### Instruments

#### Teacher Emotional Labour Strategies Scale (TELSS)

The evaluation of teachers’ emotional labour levels was done using the TELSS scale, which was designed and validated by Yin (2012). The instrument comprises 13 items assessed on a Likert scale, with each item receiving a rating ranging from one (indicating strong disagreement) to five (indicating strong agreement). It consists of three distinct sub-scales, specifically identified as surface acting (comprising six items), deep acting (encompassing four items), and the expression of naturally felt emotions (including three items). The reliability of this scale in a previous study conducted by Ghanizadeh and Royaei (2015) was .82. In the pilot test of this scale, 30 English teachers, around 16% of the population, provided responses to the questionnaire. In this piloting phase, the Cronbach alpha reliability turned out to be .62. For the full sample, the initial reliability for the 13-item scale was .54. The deletion of one poorly performing item improved the reliability to .60 for the final 12-item version. This value was considered adequate for the aims of the present study.

#### Teacher Grit Scale

The assessment of teachers’ grit was conducted using the L2 teacher grit scale, which was established and validated by Sudina et al. (2021). This instrument is in fact a 14-item Likert scale questionnaire, which is divided into two sub-scales: perseverance of efforts (comprising eight items) and consistency of interest (comprising six items). Sample items for each sub-scale are “As an ESL/EFL teacher, I am diligent” and “My level of interest in teaching ESL/EFL changes from year to year,” respectively. They are scored as one (not like me at all) to five (very much like me). Furthermore, in this scale, seven items were reversely coded. Entire calculated scores should fall between one to five (five = extremely gritty in L2 learning and one = not gritty at all in L2 learning). According to Shao (2023), the reliability coefficient of the scale, determined through the application of Cronbach’s alpha, is measured at .91. In this study, the scale demonstrated an overall reliability index of .83.

#### EFL Language Teachers’ Effectiveness Questionnaire

To gauge the English teachers’ conceptions of their success, the language teachers’ effectiveness questionnaire designed by Nayernia et al. (2022) was adopted. This questionnaire is composed of 18 five-point Likert-style items vary from one (strongly disagree) to five (strongly agree). The scale is delineated into six distinct sub-scales: oral proficiency, content and pedagogical content knowledge personality, experience, self-efficacy, and assessment literacy. The reliability index of the scale was .80 in a study by Derakhshan et al. (2022). In the current investigation, the reliability turned out to be .89. It should also be mentioned that most of the tools had already been used in the Iranian context or developed by the Iranian researchers. Nonetheless, before calculating the reliability, three EFL experts read and commented on the wording, structure, and number of items of the instruments to make it valid for the Iranian context.

#### Data Collection Procedure

To ensure the cultural and linguistic appropriateness of the instruments for the Iranian context, the questionnaires underwent a rigorous cross-cultural adaptation process. This process included forward and backward translation by independent bilingual experts, followed by a review by a panel of three professors of applied linguistics to establish content and face validity. Subsequently, the scales were pilot-tested with a sample representative of the target population to confirm their reliability and clarity. Finally, questionnaire items were put into Google Forms to collect the data. The Google Forms platform allows researchers to send their questionnaires to more people since it is more convenient and cost-efficient. A link to access the questionnaires was created and distributed among Telegram group members in which Iranian English teachers were present. Simultaneously, the researchers sent the link to English teachers they knew and asked them to circulate the link with their colleagues. A total number of 186 responses were received in about 45 days. After screening the responses, two were removed from further analysis since they seemed to be filled without enough care and attention. Thus, the remaining 184 responses were used for this study dataset. It is significant to highlight that the first section of the questionnaire included a consent form to participate in this study, demographic or factual questions including years of work experience, highest educational degree obtained, age, and gender, together with a statement that indicated the responses will remain private and only will be benefitted for research purposes. Accordingly, ethical considerations were taken into account. Following this section, three questionnaires were presented: the teacher emotional labour strategies scale, the teacher grit scale and the EFL language teachers’ effectiveness questionnaire.

#### Data analysis

Initially, the collected data was tabulated, and analysed utilising SPSS version 27. First, the dataset was screened and closely examined against the missing and outlier values. Cronbach’s alpha served as the method for assessing the reliability of the questionnaires. Initially, the researchers were determined to find a response to the research question using the structural equation modeling (SEM) technique via AMOS software. However, due to the small sample size (less than 250), choosing the SEM approach was inappropriate. Then, to evaluate the unique prediction power of each factor, multiple regression analysis was exploited through SPSS software.

## Results

Firstly, descriptive statistics were examined regarding the three variables under study: teacher emotional labour, teacher grit and teacher success in the Iranian context (*Table 1*). For each variable, the mean scores show that participants reported having different levels of emotional labour (*M* = 40.84, *SD* = 5.09), grit (*M* = 50.70, *SD* = 8.22), and teacher success (*M* = 73.80, *SD* = 8.57), respectively.

The maximum possible scores for teacher emotional labour, grit, and teacher success are 60, 70, and 90, respectively. It is evident that the average scores in all three scales are above the midpoints. In other words, all the teachers showed high levels of emotional labour, grit, and teacher success.

**Table 1:** Descriptive Statistics

	N	*M*	*SD*	Skew	Kurt	Kolmogorov-Smirnov		
						Statistics	df	Sig.
Teacher Labour Emotional	184	37.17	5.09	–.08	1.02	.09	184	<.001
Teacher Grit	184	50.70	8.225	–.23	–.26	.06	184	.06
Teacher Success	184	73.80	8.571	–1.00	3.98	.10	184	<.001

To evaluate the linear relationships between the three variables, Pearson correlation coefficients were computed. According to *Table 2*, the relationships between teacher success and teacher emotional labour, and teacher grit with teacher emotional labour were very weak and negligible. However, there was a strong, significant, and positive relationship between teacher success and teacher grit.

**Table 2:** Correlations Between the Main Variables

		Teacher Success	Teacher Emotional Labour	Teacher Grit
	Teacher success	1.000	.050	.601**
Pearson Correlation	Teacher Emotional Labour	.050	1.000	–.030
	Teacher Grit	.601**	–.030	1.000

N = 184, *p* < .05. **p* < .01.

Furthermore, the researchers checked collinearity diagnostics. Tolerance and VIF (Variance Inflation Measure) measures for the current data were .99 and 1.00, respectively. According to (Pallant, 2020), multicollinearity is indicated when the tolerance value falls below .10 and the Variance Inflation Factor (VIF) exceeds ten. Based on the results, multicollinearity was not detected. Likewise, the Mahalanobis and Cook’s values were in acceptable range. Hence, it was safe to answer the research question through regression analysis.

## Regression analysis

A standard multiple regression model was performed to check the distinct power of predicting variables in explaining the variances of scores in the outcome variable. According to *Table 3*, the results suggested that Iranian EFL teacher emotional labour and grit were significant predictors of their success, as they explained 36.6 % of the variance of their success (p < .001).

**Table 3:** Model Summary

Adjusted R Square	Std. Error of the Estimate	Change Statistics
		R Square Change
.359	6.863	.366

*Note. a. Predictors: (Constant), Teacher Grit, Teacher Emotional Labour. b. Dependent Variable: Teacher Success.*

Furthermore, the results of ANOVA in *Table 4* revealed that the model of teacher emotion labour and grit was a significant predictor of teacher success between Iranian English language teachers; F (2, 181) = 52.21, p < .001 with R2 = .36. Overall, the findings confirmed that Iranian EFL teachers with higher emotional labour and grit were more likely to succeed.

**Table 4:** The Results of ANOVA

Model	Sum of Squares	df	Mean Square	F	Sig.
Regression	4918.531	2	2459.265	52.218	.000^b^
Residual	8524.426	181	47.096		
Total	13442.957	183			

*Note. a. Dependent Variable: Teacher Success. b. Predictors: (Constant), Teacher Grit, Teacher Emotional Labour.*

To be more precise, the regression analysis (*Table 5*) verified that Iranian EFL teacher grit was a better predictor of teacher success (β = .60, t = 10.18, p = .001) than teacher emotional labour which did not reach significance (β = .06, t = 1.15, p = .25).

**Table 5:** Coeficients

	Unstandardised Coefficients		Standardised Coefficients			Correlations			Collinearity Statistics	
Model	B	Std. Error	Beta	t	Sig.	Zero-order	Partial	Part	Tolerance	VIF
(Constant)	37.25	5.22		7.12	.000					
Teacher Labour Emotional	.11	.10	.06	1.15	.25	.05	.08	.06	.999	1.00
Teacher Grit	.62	.06	.60	10.18	.000	.60	.60	.60	.999	1.00

*Note. a. Dependent Variable: Teacher Success.*

## Discussion

The central focus of the current investigation was to determine the degree of association (predictive link) between teacher emotional labour and teacher grit, which represents the perseverance and passion for enduring objectives in the context of second language (L2) teaching with teacher success. Specifically, it sought to uncover the unique power of EFL teachers’ emotional labour and their grit in further predicting the degree of teacher success in the Iranian context. The results demonstrated that teacher emotional labour and grit positively correlated with teacher success. Interestingly, emotional labour was confirmed to have a weak positive correlation with teacher success, whereas grit had a robust positive relationship with teacher success and was a significant predictor of teacher success. To put it another way, EFL teachers who reported having more grit were more successful than others having higher emotional labour.

Analogous results have been reported by Derakhshan et al. (2022), who concluded that grittier EFL teachers were more likely to enjoy the teaching profession and engage in pedagogical activities. The findings are not surprising since, in the long run, teachers develop consistency of interest and perseverance in their efforts in their careers despite many hardships and problems in L2 teaching in Iran. Similarly, Ashkani et al. (2021) found that pedagogical perceptions of EFL teachers with higher levels of grit were consistent with their real-world teaching activities and practices. The results also give credence to the outcomes and findings of Azari Noughabi et al. (2022), who found that EFL teachers with more amounts of grit and professional engagement remained committed to their profession. Additionally, the current results are congruent with that of Duckworth (2016), who highlighted the strong relationship between teaching grit and life success, which could significantly contribute to better pedagogical practice. Another major factor to consider is that grittier teachers, considering their long-term goals, are more likely to participate in professional development courses. As a result, they are much better at delivering practical instructions and possess better interpersonal communication strategies to convince their students. The results also support mindset theory of Dweck (2006) which asserts that possessing a growth attitude or mindset is normally linked to specifić beliefs about competence, emotional tendencies, and engagement. Owners of a growth mindset hold the belief that teachers’ capabilities can be ameliorated, leading them to exert substantial effort and dedicate sufficient time to enhance their skills.

Concerning teacher emotional labour, this study echoes the research insights of a previous study conducted by Burić and Frenzel (2021), who concluded that teachers who suppress or conceal their emotions are considered to provide instruction with impoverished quality. It can be inferred that hiding feelings may exhaust teachers’ mental resources and shifttheir attention away from teaching, negatively affecting their instructional behavior. The classroom is a very dynamic atmosphere, and many unpredictable events may occur. For instance, a teacher may show anger towards students who talk with each other during a lesson to stop their misbehaving. In such a case, the ability of the teacher to show fake emotions might improve their teaching and instructional skills. It may also reimburse the exhaustive mental effects such faking might have on pedagogical activities. The obtained results are in line with the Affective Events Theory, which further illustrates that professional demands in the work environment have a direct effect on work attitude, the behaviour of staff, and also their emotions and mental states, such as frustration (Weiss & Cropanzano, 1996). Positive and negative emotions are regulated by job characteristics, organisational characteristics, and individual characteristics. Similarly, Töre (2020) explored teachers’ motivation with their emotional labour and explored that there was a negative relationship between intrinsic motivation and emotional labour in Turkish teachers.

## Conclusion

This study tried to uncover the association between teacher emotional labour, grit and teacher success. The regression results testified that grit was a better and stronger predictor of teacher success than teacher emotional labour.

Teacher emotional labour has been neglected in many teacher education programmes in Iran and abroad. According to Tsang and Jiang (2018), these courses should be invigorated by adding some traces of awareness raising concerning teacher emotion and emotion regulation techniques, which are necessary to solve teachers’ emotional difficulties and to empower them. Teachers with a better sense of emotion regulation are better in their interpersonal relationships, and this could, in turn, increase their teacher credibility (Sarani et al., 2020). In addition, English teachers should be allowed to share their thoughts and experiences with their co-workers, teacher educators and head teachers. In an exciting study, Cowie (2011) found that English teachers sometimes wanted to quit their jobs or search for other work. However, they decided to continue their current teaching position since they communicated with their colleagues and found a sense of belonging to the teaching atmosphere and identity as a professional fellow. This suggests that they were receiving emotional support from others. Governing bodies, such as policymakers concerned with teachers’ psychological well-being, are recommended to consider the emotional dimension of teachers. Future researchers are suggested to check how much social support teachers receive from their colleagues and family members in relation to their emotional labour and whether it impacts their success and teaching performance. Furthermore, the role of personality factors can be investigated in tandem with grit and emotional labour. Cross-cultural comparisons with teachers from other Western and Eastern countries can also illuminate the extent to which these variables are culturally governed. Finally, it is evident that emotions are very dynamic and volatile in nature, so the utilisation of longitudinal studies could deepen our insights into the relationship between emotion, grit, and teacher success.

## Limitations

One limitation of this study was the use of self-report questionnaires for gathering data, which might increase social desirability bias. However, efforts were made to mitigate this bias by ensuring anonymity and emphasising the importance of honest responses. Meanwhile, future researchers are suggested to use multiple data collection tools or at least different qualitative methods such as focus group interviews or observations to triangulate the data and enhance the credibility of their work.

## References

[ref2] Afsharpour R., Barani G., Seyyedrezaei S.H., & Seyyedrezaei Z.S. (2024). Examining the relationship among stroke, strictness, gender identity, and teacher success from EFL teachers’ perspective. Journal of Applied Linguistics Studies, 3(1), 1–18. https://jals.aliabad.iau.ir/article_706723.html

[ref3] Al Issa H.E. (2020). When grit leads to success: The role of individual entrepreneurial orientation. Verslas: Teorija Ir Praktika, 21(2), 643–653. 10.3846/btp.2020.12346

[ref4] Al-Obaydi L.H., Shakki F., Tawafak R.M., Pikhart M., & Ugla R.L. (2023). What I know, what I want to know, what I learned: Activating EFL college students’ cognitive, behavioral, and emotional engagement through structured feedback in an online environment. Frontiers in Psychology, 13, 1083673. 10.3389/fpsyg.2022.1083673 36687994 PMC9845881

[ref5] Aronovitch A., & Gibaldi C. (2023). The importance of grit and its influence on female entrepreneurs. Entrepreneurial Business and Economics Review, 11(1 SE-Articles), 165–179. 10.15678/EBER.2023.110109

[ref6] Ary D., Jacobs L.C., Irvine C.K.S., & Walker D. (2018). Introduction to research in education. Cengage Learning.

[ref7] Ashkani P., Shabani M.B., Karimi M.N., & Esfandiari R. (2021). Correspondence between EFL teachers’ cognitive and behavioral manifestations of pedagogical beliefs: The moderating role of teacher grit. Teaching English as a Second Language Quarterly, 40(4), 35–60. 10.22099/JTLS.2021.40121.2966

[ref8] Averina F.E., & Kuswandono P. (2023). Professional development of Indonesian in-service EFL teachers: Perceived impacts and challenges. Englisia: Journal of Language, Education, and Humanities, 10(2), 71–91. 10.22373/ej.v10i2.15589

[ref9] Azari Noughabi M., Ghonsooly B., & Jahedizadeh S. (2022). Modeling the associations between EFL teachers’ immunity, L2 grit, and work engagement. *Journal of Multilingual and Multicultural Development*, 1–16. 10.1080/01434632.2022.2088766

[ref10] Benesch S. (2017). Emotions and English language teaching: Exploring teachers’ emotion labor. Routledge. 10.4324/9781315736181

[ref11] Bremner N. (2019). What makes an effective English language teacher? The life histories of 13 Mexican university students. English Language Teaching, 13(1), 163–179. 10.5539/elt.v13n1p163

[ref12] Brotheridge C.M., & Grandey A.A. (2002). Emotional labor and burnout: Comparing two perspectives of “people work.”. Journal of Vocational Behavior 60(1), 17–39. 10.1006/jvbe.2001.1815

[ref13] Buric I., & Frenzel A.C. (2021). Teacher emotional labor, instructional strategies, and students’ academic engagement: A multilevel analysis. Teachers and Teaching, 27(5), 335–352. 10.1080/13540602.2020.1740194

[ref14] Chong S.W. (2023). Language teacher autonomy and written feedback: The case of a Hong Kong elementary English teacher. In Chong S.W. & Reinders H. (Eds.), Innovation in learning-oriented language assessment (pp. 121–136). Springer International Publishing. 10.1007/978-3-031-18950-0_8

[ref15] Coombe C. (2014). 10 characteristics of highly effective EF/SL teachers. Society of Pakistan English Language Teachers: Quarterly Journal, 28(4), 2–11.

[ref16] Coombe C. (2019). Quality education begins with teachers: What are the qualities that make a TESOL teacher great? In Agudo J.D. Martínez (Ed.), Quality in TESOL and Teacher Education (pp. 173–184). Routledge. 10.4324/9780429198243-18

[ref17] Cowie N. (2011). Emotions that experienced English as a foreign language (EFL) teachers feel about their students, their colleagues and their work. Teaching and Teacher Education, 27(1), 235–242. 10.1016/j.tate.2010.08.006

[ref18] Cukur C.S. (2009). The development of the teacher emotional labor scale (TELS): validity and reliability. Educational Sciences: Theory and Practice, 9(2), 559–574.

[ref19] De Waal A., Burrell J., Drake S., Sampa C., & Mulimbika T. (2023). How to stay high-performing: developing organizational grit. Measuring Business Excellence, 27(1), 25–39. 10.1108/MBE-08-2021-0104

[ref20] Derakhshan A., Coombe C., Arabmofrad A., & Taghizadeh M. (2020). Investigating the effects of English language teachers’ professional identity and autonomy in their success. Issues in Language Teaching, 9(1), 1–28.

[ref21] Derakhshan A., Coombe C., Zhaleh K., & Tabatabaeian M. (2020). Examining the roles of continuing professional development needs and views of research in English language teachers’ success. Tesl-Ej, 24(3), 1–27.

[ref22] Derakhshan A., Dewaele J.-M., & Azari Noughabi M. (2022). Modeling the contribution of resilience, well-being, and L2 grit to foreign language teaching enjoyment among Iranian English language teachers. System, 109, 1–11. 10.1016/j.system.2022.102890

[ref23] Derakhshan A.,& Fathi J. (2024). Grit and foreign language enjoyment as predictors of EFL learners’ online engagement: The mediating role of online learning self-efficacy. The Asia-Paciffć Education Researcher, 33(4), 759–769. 10.1007/s40299-023-00745-x

[ref24] Derakhshan A., & Fathi J. (2024). Growth mindset and ideal L2 self as predictors of student engagement in EFL students: The mediating role of L2 grit. *Porta Linguarum*, .. 10.30827/portalin.viIX.29899

[ref25] Derakhshan A., & Shakki F. (2019). A critical review of language teacher education for a global society: A modular model for knowing, analyzing, recognizing, doing, and seeing. Critical Studies in Texts and Programs in Human Sciences, 19(6), 109–127. 10.30465/crtls.2019.4378

[ref26] Derakhshan A., Solhi M., & Azari Noughabi M. (2025). An investigation into the association between student-perceived affective teacher variables and students’ L2-grit. Journal of Multilingual and Multicultural Development, 46(3), 798–814. 10.1080/01434632.2023.2212644

[ref27] Duckworth A. (2016). Grit: The power of passion and perseverance. Scribner New York.

[ref28] Duckworth A.L., Peterson C., Matthews M.D., & Kelly D.R. (2007). Grit: Perseverance and passion for long-term goals. Journal of Personality and Social Psychology, 92(6), 1087–1101. 10.1037/0022-3514.92.6.1087 17547490

[ref29] Duckworth A.L., Quinn, PD., & Tsukayama E. (2021). Revisiting the factor structure of grit: A commentary on duckworth and quinn. Journal of Personality Assessment, 103(5), 573–575. 10.1080/00223891.2021.1942022 34254861

[ref30] Dweck C.S. (2006). Mindset: The new psychology of success. Random house.

[ref31] Elizabeth C.L., May C.M., & Chee P.K. (2008). Building a model to define the concept of teacher success in Hong Kong. Teaching and Teacher Education, 24(3), 623–634. 10.1016/j.tate.2007.09.007

[ref32] Ertürk G.T., Akkas F.D., & Öztürk K. (2023). Investigating foreign language ambiguity tolerance and class anxiety of preparatory level students at tertiary level. Language Education and Technology, 3(1), 1–20.

[ref33] Fan J., & Wang Y. (2022). English as a foreign language teachers’ professional success in the Chinese context: The effects of well-being and emotion regulation. Frontiers in Psychology, 13, 1–12. 10.3389/fpsyg.2022.952503 PMC9454316 36092059

[ref34] Farkhani Z.A., Badiei G., & Rostami F. (2022). Investigating the teacher’s perceptions of classroom management and teaching self-efficacy during Covid-19 pandemic in the online EFL courses. Asian-Paciffć Journal of Second and Foreign Language Education, 7(1), 25. 10.3389/fpsyg.2022.831258

[ref35] Fathi J., Mohammaddokht F., & Nourzadeh S. (2021). Grit and foreign language anxiety as predictors of willingness to communicate in the context of foreign language learning: A structural equation modeling approach. Issues in Language Teaching, 10(2), 1–30. 10.22054/ilt.2021.63362.627

[ref36] Ghanizadeh A., & Royaei N. (2015). Emotional facet of language teaching: emotion regulation and emotional labor strategies as predictors of teacher burnout. International Journal of Pedagogies and Learning, 10(2), 139–150. 10.1080/22040552.2015.1113847

[ref37] Gonzalez O., Canning J.R., Smyth H., & MacKinnon D.P. (2020). A psychometric evaluation of the Short Grit Scale: A closer look at its factor structure and scale functioning. European Journal of Psychological Assessment, 36(4), 646–657. 10.1027/1015-5759/a000535 33840984 PMC8034360

[ref38] Gu H., Mao Y., & Wang Q. (2022). Exploring EFL teachers’ emotions and the impact on their sustainable professional development in Livestream teaching: A Chinese case study. Sustainability, 14(14), 8264. 10.3390/su14148264

[ref39] Hochschild A. (1983). The managed heart: Commercialization of human feeling. University of California Press.

[ref40] Huang S. (2022). A review of the relationship between EFL teachers’ academic buoyancy, ambiguity tolerance, and hopelessness. Frontiers in Psychology, 13, 1–5. 10.3389/fpsyg.2022.831258 PMC8921681 35300165

[ref41] Kang D.-M. (2022). An elementary school EFL teacher’s emotional intelligence and emotional labor. Journal of Language, Identity & Education, 21(1), 1–14. 10.1080/15348458.2020.1777867

[ref42] Kazemkhah Hasankiadeh F., & Azari Noughabi M. (2023). Investigating the interplay among EFL teachers’ L2 grit, self-efficacy, and self-regulation: A structural equation modeling analysis. The Asia-Paciffć Education Researcher, 32(5), 707–717. 10.1007/s40299-022-00688-9

[ref43] Lee D., Reasoner K., & Lee D. (2023). Grit: What is it and why does it matter in medicine?. Postgraduate Medical Journal 99(1172), 535–541. 10.1136/postgradmedj-2021-140806 37319151

[ref44] Lee J.S. (2022). The role of grit and classroom enjoyment in EFL learners’ willingness to communicate. Journal of Multilingual and Multicultural Development, 43(5), 452–468. 10.1080/01434632.2020.1746319

[ref45] Lee J., & Xie Q. (2023). Profiling the affective characteristics of EFL learners’ digital informal learning: A person-centered approach. Innovation in Language Learning and Teaching, 17(3), 552–566. 10.1080/17501229.2022.2085713

[ref46] Li A., Hwang F., & Lu C. (2012). The development of emotional labor scales for elementary school teachers. Psychol Test, 59, 451–486.

[ref47] Li H., & Liu H. (2021). Beginning EFL teachers’ emotional labor strategies in the Chinese context. Frontiers in Psychology, 12, 1–12. 10.3389/fpsyg.2021.737746 PMC8418064 34489840

[ref48] Marashi S.M. (2023). On the relationship between reflective teaching and personality traits. Journal of English Teaching, 9(1), 108–125. 10.33541/jet.v9i1.4228

[ref49] McCain B. (2017). Effects of teacher grit on student grit and reading achievement: A mixed-methods study. Indiana University of Pennsylvania.

[ref50] Nayernia A., Nosrati R., & Mohebbi H. (2022). The factors contributing to language teachers’ effectiveness in an EFL learning context: A questionnaire validation study. Profile: Issues in Teachers’ Professional Development, 24(2), 63–79. 10.15446/profile.v24n2.93571

[ref51] Nazari M., De Costa P.I., & Karimpour S. (2023a). Novice language teacher identity construction: Similarities, differences, and beyond. Educational Linguistics, (1), 1–28. 10.1515/eduling-2022-0013

[ref52] Nazari M., De Costa P.I., & Karimpour S. (2023b). The role of institutional policy in English language teacher autonomy, agency, and identity: A poststructural perspective. *Language Teaching Research*, 13621688221143476. 10.1177/13621688221143476

[ref53] Nazari M., & Karimpour S. (2022). The role of emotion labor in English language teacher identity construction: An activity theory perspective. System, 107, 1–13. 10.1016/j.system.2022.102811

[ref54] Noor N.M., & Zainuddin M. (2011). Emotional labor and burnout among female teachers: Work–family conflict as mediator. Asian Journal of Social Psychology, 14(4), 283–293. 10.1111/j.1467-839X.2011.01349.x

[ref55] Pallant J. (2020). SPSS survival manual: A step by step guide to data analysis using IBM SPSS. Routledge. 10.4324/9781003117452

[ref56] Pishghadam R., Derakhshan A., & Zhaleh K. (2019). The interplay of teacher success, credibility, and stroke with respect to EFL students’ willingness to attend classes. Polish Psychological Bulletin, 50(4), 284–292.

[ref57] Pishghadam R., Derakhshan A., Zhaleh K., & Al-Obaydi L.H. (2023). Students’ willingness to attend EFL classes with respect to teachers’ credibility, stroke, and success: A cross-cultural study of Iranian and Iraqi students’ perceptions. Current Psychology, 42(5), 4065–4079. 10.1007/s12144-021-01738-z

[ref58] Postigo, Á., Cuesta M., García-Cueto E., Menéndez-Aller, Á., González-Nuevo C., & Muñiz J. (2021). Grit assessment: Is one dimension enough?. Journal of Personality Assessment 103(6), 786–796. 10.1080/00223891.2020.1848853 33236925

[ref59] Richards J.C. (2023). Teacher, learner and student-teacher identity in TESOL. RELC Journal, 54(1), 252–266. 10.1177/0033688221991308

[ref60] Samarasekera D.D., & Gwee M.C.E. (2020). Grit in healthcare education and practice. The Asia Pacific Scholar, 5(1), 1. 10.29060/TAPS.2020-5-1/EV5N1

[ref61] Santana-Monagas E., & Núñez J.L. (2022). Predicting students’ basic psychological need profiles through motivational appeals: Relations with grit and well-being. Learning and Individual Diffierences, 97, 1–15. 10.1016/j.lindif.2022.102162

[ref62] Sarani A., Mousapour Negari G., & Sabeki F. (2020). Educational degree, gender and accent as determinants of EFL teacher credibility. Iranian Journal of English for Academic Purposes, 8(4), 153–164.

[ref63] Sarraf M., Talepasand S., Rahimianboogar E., Mohammadifar M.A., & Najafi M. (2017). Labor as the mediator for the structural relationship between emotional job demands and teaching satisfaction and performance: The moderator role of emotional intelligence. International Journal of Behavioral Sciences, 10(4), 137–146.

[ref64] Shao G. (2023). A model of teacher enthusiasm, teacher self-efficacy, grit, and teacher well-being among English as a foreign language teachers. Frontiers in Psychology, 14, 1–16. 10.3389/fpsyg.2023.1169824 PMC10213530 37251028

[ref65] Shen Y., & Guo H. (2022). Increasing Chinese EFL learners’ grit: The role of teacher respect and support. Frontiers in Psychology, 13, 1–7. 10.3389/fpsyg.2022.880220 PMC9113390 35592152

[ref66] Sokolová, L., & Andreánska V. (2019). Pre-service teachers’ ambiguity tolerance. Society. Integration. Education. Proceedings of the International Scientific Conference, 2, 610–618. 10.17770/sie2019vol2.3676

[ref67] SoodmandAfshar H., & Doosti M. (2014). Exploring the characteristics of effective Iranian EFL teachers from students’ and teachers’ perspectives. Iranian Journal of Applied Language Studies, 6(1), 205–230. 10.22111/ijals.2014.1997

[ref68] Sudina E., Vernon T., Foster H., Del Villano H., Hernandez S., Beck D., & Plonsky L. (2021). Development and initial validation of the L2-teacher grit scale. TESOL Quarterly, 55(1), 156–184. 10.1002/tesq.581

[ref69] Tabatabaee-Yazdi M., Motallebzadeh K., Ashraf H., & Baghaei P. (2018). Continuing professional development strategies: A model for the Iranian EFL teachers’ success. SAGE Open, 8(1), 2158244018764234. 10.1177/2158244018764234

[ref70] Tahami Zarandi L., Haddad Narafshan M., & Seyfaddiny P. (2023). A comparative study of English as a foreign language teachers’ emotion regulation and well-being in Iran and Iraq. Iranian Journal of Comparative Education, 6(2), 2446–2466. 10.22034/ijce.2022.332985.1402

[ref71] Tamblyn P. (2000). Qualities of success: Lessons from a teaching career. Education Canada, 40(1), 16–19.

[ref72] Teimouri Y., Plonsky L., & Tabandeh F. (2022). L2 grit: Passion and perseverance for second-language learning. Language Teaching Research, 26(5), 893–918. 10.1177/1362168820921895

[ref73] Töre E. (2020). Effects of intrinsic motivation on teacher emotional labor: mediating role of affective commitment. International Journal of Progressive Education, 16(5), 390–403. 10.29329/ijpe.2020.277.24

[ref74] Traino K.A., Bakula D.M., Sharkey C.M., Roberts C.M., Ruppe N.M., Chaney J.M., & Mullins L.L. (2019). The role of grit in health care management skills and health-related quality of life in college students with chronic medical conditions. Journal of Pediatric Nursing, 46, 72–77. 10.1016/j.pedn.2019.02.035 30856461

[ref75] Tsang K.K., & Jiang L. (2018). Sociological understandings of teachers’ emotions in second language classrooms in the context of education/curricular reforms: directions for future research. *Emotions in Second Language Teaching: Theory, Research and Teacher Education*, 73–89. 10.1007/978-3-319-75438-3_5

[ref76] Wang Y., Derakhshan A., & Zhang L.J. (2021). Researching and practicing positive psychology in second/foreign language learning and teaching: The past, current status and future directions. Frontiers in Psychology, 12, 1–10. 10.3389/fpsyg.2021.731721 PMC8417049 34489835

[ref77] Weiss H.M., & Cropanzano R. (1996). Affective events theory. Research in Organizational Behavior, 18(1), 1–74.

[ref78] Wossenie G. (2014). EFL teachers’ self-efficacy beliefs, pedagogical success and students’ English achievement: A study on public preparatory schools in Bahir Dar Town, Ethiopia. Science, Technology and Arts Research Journal, 3(2), 221–228. 10.4314/star.v3i2.29

[ref79] Wróbel M. (2013). Can empathy lead to emotional exhaustion in teachers? The mediating role of emotional labor. International Journal of Occupational Medicine and Environmental Health, 26, 581–592. 10.2478/s13382-013-0123-1 24057262

[ref80] Yang C., & Chen A. (2021). Emotional labor: A comprehensive literature review. Human Systems Management, 40, 479–501. 10.3233/HSM-200937

[ref81] Yin H. (2012). Adaptation and validation of the teacher emotional labor strategy scale in China. Educational Psychology: An International Journal of Experimental Educational Psychology, 32(4), 451–465. 10.1080/01443410.2012.674488

[ref82] Yin H., Huang S., & Lee J.C.K. (2017). Choose your strategy wisely: Examining the relationships between emotional labor in teaching and teacher efficacy in Hong Kong primary schools. Teaching and Teacher Education, 66(1), 127–136. 10.1016/j.tate.2017.04.006

[ref83] Zhang L.J., Fathi J., & Mohammaddokht F. (2023). Predicting teaching enjoyment from teachers’ perceived school climate, self-efficacy, and psychological wellbeing at work: EFL teachers. Perceptual and Motor Skills, 130(5), 2269–2299. 10.1177/00315125231182269 37395156 PMC10552353

[ref84] Zhi R., Wang Y., & Wang Y. (2024). The role of emotional intelligence and self-efficacy in EFL teachers’ technology adoption. The Asia-Paciffć Education Researcher, 33, 845–856. 10.1007/s40299-023-00782-6

